# Tomato pomace as a source of valuable functional ingredients for improving physicochemical and sensory properties and extending the shelf life of foods: A review

**DOI:** 10.1016/j.heliyon.2024.e25261

**Published:** 2024-01-29

**Authors:** Ifagbémi Bienvenue Chabi, Oscar Zannou, Emmanuelle S.C.A. Dedehou, Bernolde Paul Ayegnon, Oloudé B. Oscar Odouaro, Sajid Maqsood, Charis M. Galanakis, Adéchola Pierre Polycarpe Kayodé

**Affiliations:** a Laboratory of Human Nutrition and Valorization of Food Bio-Ingredients, Faculty of Agricultural Sciences, University of Abomey-Calavi, 03 BP 2819, Jericho Cotonou, Benin; b Ecole des Sciences et Techniques de Conservation et de Transformation des Produits Agricoles, Université Nationale d’Agriculture (UNA), BP 114, Sakété, Benin; c Food Science Department, College of Agriculture and Veterinary Medicine, United Arab Emirates University, Al-Ain, 15551, United Arab Emirates; d National Water and Energy Center, United Arab Emirates University, Al-Ain, 15551, United Arab Emirates; e Research & Innovation Department, Galanakis Laboratories, Chania, Greece; f Food Waste Recovery Group, ISEKI Food Association, Vienna, Austria; g College of Science, Taif University, Taif, Saudi Arabia

**Keywords:** Antioxidant, Bioactive compounds, Health benefits, Shelf life, Sensory properties, Tomato pomace

## Abstract

Due to its nutritional and bioactive content, tomato pomace (TP) remains among the world's richest fruits and vegetables. Tomatoes and TP (generated coproduct) are a very rich source of lycopene and other carotenoid compounds and contain an essential amount of polyphenols, policosanol, phytosterols, organic acids, dietary fibers, minerals, and vitamins. TP is a promising source of significant bioactive compounds with antioxidant and antimicrobial potential. Therefore, their consumption is known to be effective in preventing certain chronic diseases. For example, lycopene prevents prostate cancer and acts as a hepatoprotector and genoprotector against mycotoxins, pesticide residues, and heavy metals. Thus, the valorization of TP as a food ingredient can be of great health, economic and environmental interest and contribute to improving nutrition and food security. During the last decades, considerable efforts have been made to valorize TP as a crucial functional ingredient in improving: (i) the nutritional and functional properties, (ii) sensory characteristics and (iii) the shelf life of many foods. The current review aims to update and summarize the knowledge on the recent food applications of TP, particularly its use as a functional ingredient to improve the functional properties and shelf life of foods.

## 1 Introduction

**Fig. 1:**
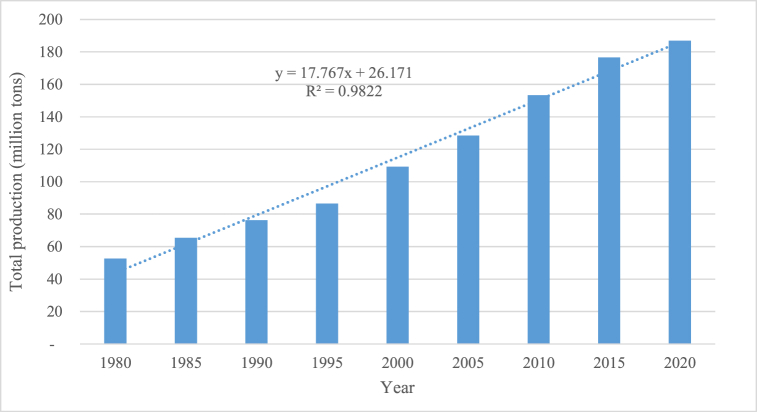
Changes in world tomato production since 1980.

The tomato (*Lycopersicon esculentum*) is one of the most widely produced vegetables in the world. It is an essential food commodity in the human diet, and its production is constantly increasing. According to Fig. 1, world tomato production has evolved steadily since 1980.2021, approximately 189 million tons of tomatoes were produced [1]. Based on the estimated growth rate (3.22) from 1980 to 2020 (Fig. 1), world production is estimated to be around 222 million tons by 2030.

Tomato is an essential source of micronutrients such as provitamin A, vitamin C, vitamin E, minerals as well as providing significant amounts of bioactive compounds. It is, therefore, a functional food widely consumed by people worldwide, both in raw and cooked form, or used as a raw material for processing into products such as fruit juice, puree, sauces, ketchup, paste, and tomato powder. In recent decades, its consumption has increased significantly. This rise is associated with the health benefits induced by its numerous bioactive molecules. Indeed, tomato contains major phytochemical compounds such as tetraterpenoids, polyphenols, tools, ascorbic acid, policosanol, and phytosterols, known for their preventive and curative effects against several chronic diseases. These diseases include prostate and skin cancers, obesity, type 2 diabetes, atherosclerosis, cardiovascular diseases as well as bowel disorders and cognitive impairment [[2], [3], [4], [5], [6], [7]].

**Fig. 2:**
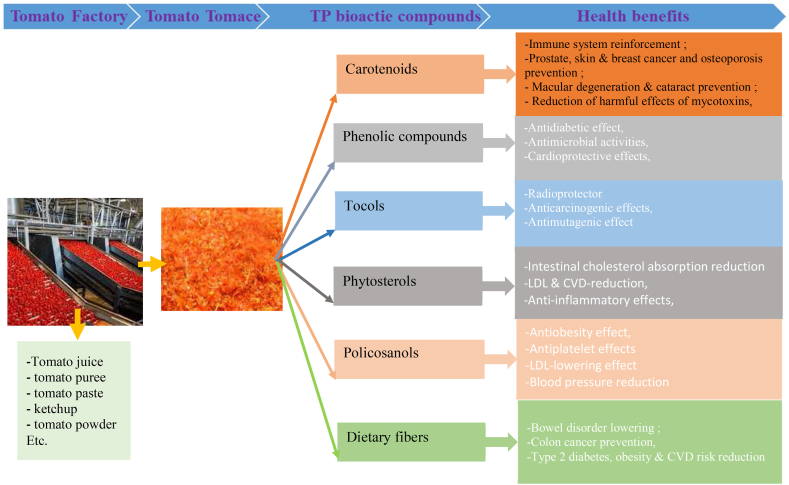
Health benefits of the main bioactive compounds of TP.

Approximately one-third of the total tomato production is processed yearly [3]. Tomato industrial processings generate wet waste called tomato pomace (TP). This latter comprises peel, pulp, and seeds, accounting for nearly 5–30 % (w/w) of the initial raw material [8,9]. Thus, every year, about 5.4–9.0 million tons of TP are produced by food industries worldwide [6]. Therefore, managing this waste has always been a significant concern for the food industries. Primarily, TP was disposed of in landfills, used as a cheap source of fertilizer for the soil, or used for livestock feed [9,10]. Yet, like whole tomato, the differents parts of the TP are also rich in macro and micronutrients and contain significant amounts of various bioactive molecules (Fig. 2). For example, lycopene is one of the most represented and the most active molecule in tomatoes. It is more critical in TP (especially in peels, up to 864 mg/kg) than in whole tomatoes [[11], [12], [13]]. Recently, lycopene has significantly reduced natural and chemical toxins [14,15]. Lycopene consumption decreased the harmful effects of some mycotoxins, especially Aflatoxins B1, M1, and P1, and ochratoxins [14,15]. It has also been reported that lycopene acts as a hepatoprotector and genoprotector against pesticides and heavy metals. Recent epidemiological research revealed the protective effects of bioactive tomato molecules, especially carotenoids and polyphenols, against two major chronic diseases, type 2 diabetes and obesity [7]. In addition, lycopene and other bioactive molecules from TP exhibit antimicrobial activities against a wide range of pathogens, including multi-drug-resistant microorganisms [14,15]. There is, therefore, great potential for recovering these valuable bioactive molecules from TP or its different parts. They have industrial applications in the food, cosmetic, and pharmaceutical industries [3]. Various food applications of TP, including its bioactive compounds, have been widely reported in the literature. Notably, studies have been conducted on adding TP or part of its constituents as a functional ingredient to improve the physicochemical and sensory properties and extend the shelf life of foods [[16], [17], [18], [19], [20]]. In this review, we have summarized the most recent developments in the food valorization of TP, especially the effect of pomace or some of its constituents on the physicochemical properties and shelf life of foods. We have also highlighted its influences on the organoleptic properties of foods.

## 2 Data collection methodology

An online thorough, comprehensive literature search was carried out by combining the relevant keywords, including “tomato,” “tomato pomace,” “*Lycopersicon esculentum*,” “nutritional composition,” “proximate composition,” “food application,” “food ingredient,” “shelf life,” “bioactive compounds,” “antioxidant properties,” “sensory properties,” “health benefits.” Regarding the search engine, Google, Google Scholar, ScienceDirect, Web of Science, Directory of Open Access Journals, Scopus, SpringerLink, Pubmed, and Wiley Online Library were independently scrutinized. In addition, articles published in both English and French were considered. The final search was performed on April 30, 2023. References were managed with Mendeley software.

## 3 Nutritional composition of tomato fruit

### 3.1 Proximate composition

Nutritionally, the compositional analysis of tomato components revealed the presence of both macronutrients and micronutrients with significant variations. Fresh tomato fruit provides protein, lipid, carbohydrate, and dietary fibers concerning macronutrients. In recently published studies, the ranges of the proximate composition of fresh tomatoes consist of 68.03–96.17 g/100 g of moisture content, 5.90–10.60 % of ash, 10.50–25.03 g/100 g of total protein, 3.62–5.39 g/100 g of lipid, 3.92–8.00 g/100 g of carbohydrates, 47.00–56.45 g/100 g of total sugar, 3.61–4.08 of pH, 0.39–0.55 % of acidity, 30.03–41.21 % of reducing sugar and 18.00–75.00 kcal/100 g of energy [5,[21], [22], [23], [24], [25], [26]]. Water is the most crucial constituent in fresh tomato fruit, and the quantity depends on the tomato varieties, especially the environmental conditions in which it is cultivated. This abundance of water in tomatoes renders them one of the most highly perishable products. However, the contents of tomatoes in protein, lipid, and carbohydrates suggest that they are richer in macronutrients than pepper [27]. Fresh tomatoes have an average fructose, glucose, sucrose, and total fiber content of 2.88 %, 2.45 %, 0.02 %, and 11.44 g/100 g, respectively [28]. The by-products of tomatoes are valorized for multipurpose thanks to their contents of diverse nutrients. The proximate composition of the peels is constituted of moisture, protein, carbohydrate, lipid, acidity, pH, and energy, which have ranges of values as 5.71–90.34 g/100 g, 9.93–23.25 g/100 g, 6.01–64.35 g/100 g, 4.06–16.73 g/100 g, 2.43–5.80 %, 3.59–5.98 and 200.07–344.03 kcal/100 g, respectively [3,29]. The tomato seeds also add value regarding their physicochemical composition, displaying the moisture, protein, carbohydrate, lipid, acidity, pH, and energy of 12.97–22.42 g/100 g, 12.94–16.19 g/100 g, 10.60–38.50 g/100 g, 18.32–22.62 g/100 g, 4.67–5.66 g/100 g, 5.76–6.08 and 269.96–400.59 kcal/100 g, respectively [29]. As evidenced, the peels display protein, carbohydrate, and lipid values, making them more valuable than the seeds. The TP is widely valorized for a wide range of food products. Its moisture, protein, fat, dietary fiber, and ash composition is found in 62.27–70.14 %, 16.81–23.25 g/100 g, 11.17–16.73 g/100 g, 48.62–53.97 g/100 g, and 3.33–4.02 g/100 g, respectively [30].

### 3.2 Mineral composition

Minerals play pivotal roles in regulating metabolic pathways, forming vital organs, maintaining bodily physiological functions, regulating pH balance, fluid balance, blood pressure, nerve transmission, muscle contraction, and energy production [28,31,32]. The mineral composition of tomato and its by-products is shown in Table 1. Studies showed that the most essential minerals in tomatoes are potassium (K), phosphorus (P), calcium (Ca), and magnesium (Mg). Other minor minerals, including iron (Fe), Zinc (Zn), copper (Cu), sodium (Na), boron (B), and manganese (Mn), have also been reported in tomatoes Table 1. It has been found that the level of repining [33] and farming conditions [34] influence the mineral composition of tomatoes. As displayed in Table 1. TP, especially peels and seeds, are valuable sources of minerals such as K, P, Ca, Mg, Fe, and Na [3,21,33,[35], [36], [37], [38]]. Isik and Yapar [36] reported that tomato by-products like tomato seeds could reinforce tarhana in micronutrients like minerals. Likewise, Mehta and colleagues [37] revealed that incorporating TP in bread and muffin enriched these foods with minerals such as P, Ca, Mg, Fe, and Na.

**Table 1:** Mineral composition of tomato and tomato by-products ([3,21,[33], [34], [35], [36], [37], [38]]).

Mineral composition (μg/g)	Tomato fruit	Tomato peels	Tomato pulp	Tomato seeds	TP
P	160–5160	2620.0–3430	1.5–4	10737.6	–
K	900–62920	10970–45830	23–42	9765.3	2686.9
Fe	1.81–317.58	15–56.2	16–32	240.9	29.2
Zn	1.34–40.35	5.30–39.02	17–42	96.8	–
B	25.84–55.64	25.84–48.59	4.5–16.5	–	–
Cu	0.39–11.39	2.44–11	1.0–5.8	18.8	–
Mg	64.5–2655.9	1356.1–2655.9	0.75–1.5	5037.1	281.3
Ca	70.8–6020	398.3–1600.	0.60–1.25	1347.6	146.40
Mn	0.1–3.6	0.5–14.	5–13	77.7	
Na	20.4–320	720–783.3	–	–	210.3
S	620–830		0.8–2.0		

### 3.3 Vitamin composition

Vitamins are essential micronutrients with specific physiological functions, including enzyme cofactors, biological antioxidants, hormones, and photoreceptive cofactors in vision in the human body [39]. Tomatoes are rich in vitamins A, E, C, niacin, folate, B1, B2, B12, K, and H (Table 2), essential to human health [24,40]. Vitamin C, which is necessary for the immune system strengthening, is also found in tomato peels, pulp, seeds, and pomace. In addition, tomato seeds contain niacin and vitamin E, which TP is a rich source of vitamins B12 and C [28,41,42]. Tomato and its by-products can help alleviate many diseases due to high concentrations of vitamin C, E, and other bioactive compounds [39].

**Table 2:** Vitamins composition of tomato and tomato by-products.

Vitamin composition (mg/100 g)	Tomato fruit	Tomato peels	Tomato pulp	Tomato seeds	TP	References
A	0.004–0.010	–	–	28.20	–	[24,29,[40], [41], [42],^54^,^68^,[118], [119], [120]]
C	5.71–101.29	110.00	16.80–19.30	9.50	14.03	
B1	0.216	–	–	1370.00–2340.00	–	
B2	0.25	–	–	–	–	
B6	0.47	–	–	–	–	
E	0.42–3.26	–	–	0.56–32.7	–	
Folate	13	–	–	–	–	
B12	0.00004–0.00005	–	–	–	1110.00	
Niacin	0.98–1.11	–	–	0.6	–	
K	0.0013–0.00149	–	–	–	–	
H	0.00047–0.00068	–	–	–	–	

### 3.4 Amino acid composition

Amino acids are the building blocks of proteins that maintain body cells, regulate muscle protein metabolism, control growth and immunity, wound healing, reduce adiposity, and repair damaged tissues [28]. The primary amino acids of tomato fruit are aspartic acid, glutamic acid, glutamine, alanine, phenylalanine, histidine, serine, cysteine, valine, threonine, and proline (Table 3). However, the amino acid content in tomatoes depends on the cultivation conditions [43,44] and maturation and ripening [45,46]. For instance, Zhu and colleagues [43] reported that the Se treatment of pink tomato with selenium increased the content of amino acids and the amounts of soluble sugars and bioactive compounds. Moreover, Sun and colleagues [44] found out that the content of amino acids was affected in tomatoes treated with L-glutamate. Tomato seeds are the richest tomato by-product in amino acids compared to peels and pomace (Table 3). The seeds are rich in glutamic acid, aspartic acid, arginine, leucine, glycine, alanine, phenylalanine, lysine, serine, and proline (Table 3). Furthermore, tomato peels and pomace are rich sources of glutamic acid, aspartic acid, arginine, leucine, isoleucine, alanine, and glycine (Table 3) [44,[47], [48], [49]]. Regarding their amino acid content, tomato by-products can reinforce foods low in amino acids, such as cereal-based foods [50].

**Table 3:** Amino acid composition of tomato and tomato by-products.

Amino acid composition	Tomato fruit	Tomato peels	Tomato seeds	Tomato pomace	References
Leucine	0.24–1.41 mg/100 g f.w; 24.50–590.00 mg/100 g d.w; 26.60–134.60 nmol/g f.w	380.00 mg/100 g d.w	780.00 mg/100 g d.w	1070.00 mg/100 g d.w	[44,[47], [48], [49]]
Valine	0.84–2.15 mg/100 g f.w; 34.06–893.00 mg/100 g d.w; 56.00–216.00 nmol/g f.w	320.00 mg/100 g d.w	560.00 mg/100 g d.w		
Lysine	0.05–13.00 mg/100 g f.w; 25.58–120.00 mg/100 g d.w; 170.60–549.00 nmol/g f.w	140.00 mg/100 g d.w	610.00 mg/100 g d.w	880.00 mg/100 g d.w	
Isoleucine	0.42–1.41 mg/100 g f.w; 15.81–186.00 mg/100 g d.w; 1200.00–6397.00 nmol/g f.w	230.00 mg/100 g d.w	460.00 mg/100 g d.w	690.00 mg/100 g d.w	
Threonine	0.67–2.84 mg/100 g f.w; 14.00–104.32 mg/100 g d.w	240.00 mg/100 g d.w	450.00 mg/100 g d.w	550.00 mg/100 g d.w	
Histidine	0.07–5.50 mg/100 g f.w; 18.70–99.00 mg/100 g d.w; 108.00–389.00 nmol/g f.w	70.00 mg/100 g d.w	260.00 mg/100 g d.w		
Phenylalanine	0.38–23.00 mg/100 g f.w; 10.00–162.99 mg/100 g d.w; 51.20–144.00 nmol/g f.w	260.00 mg/100 g d.w	540.00 mg/100 g d.w	610.00 mg/100 g d.w	
Methionine	0.41–1.20 mg/100 g f.w; 10.34–616.00 mg/100 g d.w; 3653.00–5600.00 nmol/g f.w	90.00 mg/100 g d.w	190.00 mg/100 g d.w	270.00 mg/100 g d.w	
Tryptophan	240.00–880.00 nmol/g f.w				
Aspartic acid	0.92–30.00 mg/100 g f.w; 69.00–816.00 mg/100 g d.w; 6.10–27.30 nmol/g f.w	1230.00 mg/100 g d.w	1510.00 mg/100 g d.w	1570.00 mg/100 g d.w	
Arginine	0.08–9.00 mg/100 g f.w; 22.70–81.50 mg/100 g d.w; 74.60–146.60 nmol/g f.w	1230.00 mg/100 g d.w	1510.00 mg/100 g d.w	1460.00 mg/100 g d.w	
Glycine	0.12–0.90 mg/100 g f.w; 5.54–310.00 mg/100 g d.w; 541.00–2421.00 nmol/g f.w	370.00 mg/100 g d.w	720.00 mg/100 g d.w	630.00 mg/100 g d.w	
Glutamic acid	1.62–180.00 mg/100 g f.w; 51.00–3551.00 mg/100 g d.w; 34.6–84.00 nmol/g f.w	3850.00 mg/100 g d.w	2950.00 mg/100 g d.w	7210.00 mg/100 g d.w	
Proline	0.09–1.56 mg/100 g f.w; 43.11–1884.00 mg/100 g d.w; 60.50–218.60 nmol/g f.w	230.00 mg/100 g d.w	530.00 mg/100 g d.w		
Cysteine	53.30–149.00 nmol/g f.w	70.00 mg/100 g d.w	210.00 mg/100 g d.w	230.00 mg/100 g d.w	
Serine	0.09–10.62 mg/100 g f.w; 18.00–135.30 mg/100 g d.w; 0.66–1.44 nmol/g f.w	250.00 mg/100 g d.w	560.00 mg/100 g d.w	170.00 mg/100 g d.w	
Alanine	0.18–5.31 mg/100 g f.w; 15.9–90.30 mg/100 g d.w; 704.00–1653.00 nmol/g f.w	380.00 mg/100 g d.w	600.00 mg/100 g d.w	710.00 mg/100 g d.w	
Tyrosine	0.13–1.27 mg/100 g f.w; 12.00–24.10 mg/100 g d.w; 226.60–413.30 nmol/g f.w			690.00 mg/100 g d.w	
Asparagine	1.74–9.13 mg/100 g f.w; 21.55–246.00 mg/100 g d.w; 349.30–1816.00 nmol/g f.w				
Glutamine	15.00–36.30 mg/100 g f.w; 54.00–1604.00 mg/100 g d.w; 108.00–986.60 nmol/g f.w				
GABA	10.50–12.00 mg/100 g f.w; 174.00–735.10 mg/100 g d.w				

### 3.5 Fatty acid composition

The nutritional values of tomato and tomato by-products include many fatty acid types (Table 4). Primary fatty acids in tomato and tomato peels, pulp, seeds, and pomace are linoleic acid, oleic acid, palmitic acid, stearic acid, and linolenic acid. Some fatty acids are also present in tomato and tomato by-products at low concentrations compared to primary fatty acids. These fatty acids include tridecanoic acid, myristic acid, palmitoleic acid, pentadecanoic acid, margaric acid, elaidic acid, octadecadienoic acid, arachidic acid as well as eicosadienoic acid, behenic acid, tricosanoic acid and tetracosanoic acid (Table 4). Fatty acids are vital in the human body as modulators of membranes, energy suppliers, and storage material [38]. The essential fatty acids are considered functional food and nutraceuticals having cardioprotective, antiatherogenic, antithrombotic, anti-inflammatory, antiarrhythmic, hypolipidemic, anticancer, and antidiabetes [[51], [52], [53]]. The composition and content of fatty acids in tomatoes depend on the cultural environment, variety, storage conditions, and ripening level. For instance, Khan and colleagues [54] reported that the increase in atmospheric carbon dioxide in the air affects not only the fatty acid content but also the proximate composition, elemental concentration, and vitamin C contents of tomatoes. Moreover, Saini and colleagues [9] reported that significant increases in polyunsaturated fatty acids, carotenoids, and α-tocopherol induce tomato ripening.

**Table 4:** Fatty composition of tomato by-products.

Fatty composition	Tomato peels (mg/100 g)	Tomato peels (mg/100 g)	Tomato pulp (mg/100 g)	Tomato seeds (mg/100 g)	Tomato pomace (mg/100 g)	References
Tridecanoic acid	510.00–2030.00	–	–	–	–	[ [21,47,54]]
Myristic acid	1.00–360	340.00	–		410.00	
Pentadecanoic acid	1.00–2.00	–	–		90.00	
Pentadecenoic acid	–	–	–		90.00	
Palmitic acid	46.00–17700.00	15190.00	251.20–713.90	943.80–2371.30	16320.00	
Palmitoleic acid	2.00	1820.00	–	–	640.00	
Heptadecanoic acid	1.00–3.00	–	–	–	190.00	
Heptadecenoic acid	–	–	–	–	520.00	
Stearic acid	10.00–6240.00	6840.00	57.00–121.20	120.90–762.00	5430.00	
Oleic cis	5.00–26600.00	19140.00	39.10–198.30	202.30–3053.10	18500.00	
Elaidic acid	1.00–7.00					
Cis linoleic acid	7.00–49300.00	52410.00	335.90–1419.30	2064.20–9325.50	51910.00	
Trans linoleic acid	2.00–4.00					
Α-Linolenic acid	63.00–5610.00	4260.00	208.80–548.80	403.50–729.60	3350.00	
Octadecatetraenoic acid					480.00	
Eicosadienoic acid	6.00				150.00	
Eicosatrienoic acid					70.00	
Docosadienoic acid					390.00	
Docosatrienoic acid					550.00	
Docosatrienoic acid					130.00	
Tricosanoic acid	1.00–4.00					
Tetracosanoic acid	2.00–7.00					
Behenic acid	1.00–300.00		8.30–20.70	24.00–41.40		
Eicosapentaenoic					260.00	
Lignoceric	650.00		6.30–22.90	31.00–42.10	290.00	
Arachidic acid	3.00–630.00		21.30–50.30	75.50–125.00		

## 4 Bioactive compounds

Bioactive compounds present in tomatoes and their TP include tetraterpenoids (α and β carotene, lycopene, lutein, zeaxanthin, β-cryptoxanthin), tocols (tocopherols and tocotrienols), polyphenols (phenolic acids, flavonoids), policosanol, canolols, phytosterols and dietary fibers (Fig. 2). These various phytochemicals are unevenly distributed in the different parts of the vegetable. The most representative phytochemicals in TP are carotenoids, polyphenols, tocols, and phytosterols. The others (policosanol, canolols, and phytosterols) are less represented, although they ensure no less critical biological functions.

### 4.1 Carotenoids

Carotenoids are one of the main constituents of TP. Nagarajan and colleagues [55] reported a total carotenoid content of 9.37–10.81 mg/100 g for wet samples. Vági and colleagues (2007) recorded the total carotenoid content of dried TP to be 34.86–195.99 mg/100 mg/100 g using supercritical carbon dioxide as an extraction medium. Among carotenoids, TP has a higher concentration of β-carotene, estimated between 3.40 and 5.55 mg/100 g [56,57]. Lycopene is a pigment found mainly in tomato peels that deteriorates quickly upon exposure to light, although it is resistant to thermal processing [58]. Lycopene content in TP varies greatly depending on the tomato variety and extraction conditions. The lycopene content of peels is two and a half and five times greater than that of seeds and pulp, respectively [9,19]. Its concentration in the peels can reach 288 mg/100 g [6]. In addition, it has shown highly potent antioxidant activity, one hundred times greater than tocols [59]. Palomo and colleagues [57] and Concha-Meyer [60] reported a lycopene concentration of 0.001–7.74 mg/100 g, which is low compared to the β-carotene content reported in dried TP. In contrast, Vági and colleagues [61] proved that the percentage of lycopene in the carotenoid-rich extract was 70–90 %, making lycopene the most valuable compound among the others in TP with a concentration of 31.40 mg/100 g. Zhang and colleagues [62] reported a lycopene content in freeze-dried pomace to be dry at 32 g/100 g, while Eh and Teoh [63] determined a 5.11 mg/g by applying ultrasound-assisted extraction. Recently, a study demonstrated that both drying methods of TP and extraction techniques influence the yield of lycopene, which could vary from 5.66 to 59.66 μg/g [64]. Other carotenoids include lutein, neo-lutein, α-cryptoxanthin, β-cryptoxanthin, lycoxanthin, *cis*-lycoxanthin, lycopene, and neo-lycopene [61].

### 4.2 Phenolic compounds

TP is a source of phenolic compounds such as phenolic acids and flavonoids. The total phenolic content of TP is determined between 0.94 mg GAE/g and 1.03 mg GAE/g as affected by the extraction techniques [65]. The microwave-assisted extraction technique's total phenolic content was 2.85–17.87 mg GAE/g, whereas the total phenolic content for ultrasound-assisted extraction was between 1.81 and 18.65 mg GAE/g [66]. Eight phenolic compounds were identified in TP, including the phenolic acids and flavonoids commonly found in tomatoes [65,67,68]. They include chlorogenic acid, caffeic acid, rutin, isoquercetin, quercetin, ferulic acid, gallic acid and naringenin. Bao and colleagues [65] reported that TP's major phenolic compounds are gallic acid, chlorogenic acid, naringenin, and rutin. Further phenolic compounds, including *p*-coumaric acid, kaempferol 3-O-glucoside, and (+)-catechin, have been identified in TP extracted with deep eutectic solvents [69,70]. In addition, Abbasi-Parizad and colleagues [71] reported besides the above-cited compounds, 31.50, 12.90, 59.70, 32.30, 98.00, and 14.70 μg/g of vanillic acid, syringic acid, *p*-coumaric acid, sinapic acid, myricetin, and apigenin, respectively.

### 4.3 Tocopherols and phytosterol

Additional bioactive molecules have been found in the TP, including tocopherol, health-preventive phytosterol, and vitamin C, which are often extracted with lycopene and other hydrophilic antioxidant molecules, such as flavonoids and phenolic acids [71]. Tocopherols are mainly localized in tomato seeds. The individual tocopherols, including α-tocopherol, γ-tocotrienol, γ-tocopherol, and δ-tocopherol, have been identified in TP with concentrations of 88.00–3600.00, 96.00–1655.00, 17.00–922.00 and 11.00–913.00 μg/g, respectively [61,72]. Regarding the β-sitosterol, the contents in TP ranged between 0.68 and 6.21 mg/100 g dry material [61]. Moreover, campesterol (1359.00 μg/g), stigmasterol (2027.00 μg/g), and β-sitosterol (8236.00 μg/g) have been discovered in TP [72].

### 4.4 Other bioactive molecules

Among the less-represented biomolecules of TP, policosanol is getting more and more attention. It is a chemical compound consisting of various long-chain aliphatic fatty alcohols. It was initially found in natural sources such as sugarcane and beeswax. Recently, it has been identified in plant products such as cereals, fruits, leaves, seeds, nuts, and animal products [2]. TP, especially tomato seed oil, contains a significant amount of policosanol (64–72 mg/kg). Several extraction methods, including conventional (Solvent extraction, saponification, transesterification) and emerging extraction methods (Supercritical CO_2_, ultrasonic-assisted, microwave-assisted, and pressurized solvent), enhance the extraction of policosanol from its matrix. Among these, Shen and colleagues concluded that the supercritical CO_2_ extraction method is the best in terms of purity, product yield, and extraction time [73]. However, it is a costly extraction method.

Besides, despite its high unsaturated fatty acids (80 %) content, tomato seed oil demonstrated excellent heat (180 °C for 50 h) stability [74]. Bioactive compounds of this oil, especially tocols and polyphenols, ensure this thermal stability. Canolol is the only known lipophilic phenolic compound formed from the hydrolysis followed by decarboxylation of sinapine during processing. It was reported that roasting the seeds before extraction resulted in a significant increase (up to 100-120-fold) in canolol formation compared to unroasted seeds [75,76]. As a lipophilic phenolic compound, canolol is a critical biomolecule responsible for the thermal stability observed with tomato seed oil [76].

## 5 Health benefits of TP bioactive compounds

### 5.1 Health benefits of TP

The main health-promoting effects of TP phytochemicals (carotenoids, tocols, polyphenols, and sterols) are their potent antioxidant activities and, consequently, their protective effects against chronic diseases are related to the oxidative effect. The primary health benefits of TP biomolecules are displayed in Fig. 2. In addition, it has been reported that TP possesses more significant biological effects than tomatoes. For instance, Widjaja and colleagues showed that TP contains powerful antithrombotic activities, even more important than tomatoes [77]. In addition, a recent clinical study showed that the daily oral intake for five days of 1 g of TP aqueous extract containing nucleosides and polyphenols inhibited human platelet aggregation [57].

Moreover, hamsters fed diets enriched with TP (42 %), defatted tomato seeds (18 %), and tomato seeds (10 %) showed a reduction in total cholesterol levels [78]. These authors concluded that the content of bioactive protein, dietary fiber, and polyphenols of different tomato by-products was responsible for reducing total cholesterol levels. Dietary fibers are known for their satietogenic effects in humans. Recent recommendations indicate a daily intake of 25 g and 38 g for adult women and men [6]. Additionally, dietary fiber and polyphenols are widely cited in the literature as having important antioxidant and anti-inflammatory effects and exert significant preventive and curative effects against certain chronic diseases, including obesity, type 2 diabetes, and cardiovascular disease [6]. Likewise, policosanol is another relevant lipophilic biomolecule of TP. It has a potent antioxidant and exhibits antimicrobial and antidermatophytic properties against pathogen germs [73]. Several studies reported the multiple biological properties of policosanol against chronic diseases, including type 2 diabetes, obesity, aging, cerebrovascular disease, type 2 hypercholesterolemia, and Parkinson's disease [73,79]. According to these authors, the daily dose recommended ranges between 5 and 80 mg/d depending on whether one wants to lower serum cholesterol levels or treat type 2 diabetes or others. Other bioactive compounds, such as phytosterols and canolols, have also shown significant health effects. For example, phytosterols are intensely involved in preventing and treating certain chronic hormone-dependent diseases. In addition, they contribute to the reduction of plasma LDL levels [78]. As for canolol, it is highly potent in free radical scavenging and exhibits antimutagenic activity against the action of Salmonella [76]. Its anticancer effects are also reported.

Lycopene is the primary bioactive compound of tomato pomace. The investigations showed that it has tremendous health benefits.

### 5.2 Health benefits of lycopene

Like all compounds of the tetraterpenoid family, Lycopene plays a pivotal biological role in human well-being. Some studies have reported that lycopene has anti-inflammatory and cardioprotective activities and may also exhibit biological effects in certain chronic diseases, including cancers, diabetes, obesity, and other metabolic syndromes [80,81]. In addition, lycopene shows potent biological effects against harmful agents like mycotoxins, heavy metals, pesticides, and other natural toxins [59]. However, it is essential to note that the presence of tomato seed oil followed by heat treatment (pasteurization) significantly improves the bioaccessibility of lycopene and other carotenoids and increases the positive digestion of lipids [82]. This could be one of the most relevant ways to maximize the bioactivity of carotenoid compounds in humans. Several studies have reported the preventive role of lycopene in the risk of prostate cancer development and its protective properties against hepatotoxicity and genotoxicity induced by various agents.

#### 5.2.1 Prevention of prostate cancer

Clinical and epidemiological research has shown an inverse association between consuming foods containing lycopene and the risk of prostate cancer. Moreover, most *in vivo* studies have confirmed the anticancer activities of lycopene, particularly in prostate cancer [83]. For Mirahmadi et al. [84], lycopene can exert its anticancer effect through various pathways: by blocking the gap junction molecules and inhibiting the colony formation, decreasing the motility, increasing the anti-oxidative and anti-proliferative effects, reducing inflammatory cytokines, etc. For example, Jiang et al. [85] have demonstrated that the anticancer effect of lycopene is linked to its role in suppressing inflammatory responses. Soares et al. [86] observed that lycopene extracted from tomato sauce and ketchup promoted a decrease in some prostate cancer cells. The antioxidant properties of lycopene should help to neutralize reactive oxygen species, thereby reducing oxidative stress and tumor progression. However, when combined with vitamin E or docetaxel, the preventive effect of lycopene on prostate cancer is more effective [87].

#### 5.2.2 Protection against hepatotoxicity

Hepatotoxicity poses a significant threat to liver health. It results from exposure to various chemical, medicinal, or toxic agents. However, lycopene is known to have a critical protective effect against many toxins, including bacterial toxins and some mycotoxins [59]. For the same authors, the combination of lycopene with vitamin E has a significative effect against aflatoxin toxicity. It has also been reported that tomato lycopene has an inhibition action on the impact of some bacterial toxins such as bacterial lipopolysaccharide, microcystin-LRs, and other toxins like D-galactosamine [59,88]. According to some authors, Lycopene also has a substantial protective effect on various chemicals and metals such as cadmium, copper, mercury, lead, etc. [89,90]. Hedayati et al. [59] link the protect effect of lycopene against hepatotoxicity to its anti-oxidative, free-radical scavenging, chelating, and anti-apoptotic properties. All of these have the potential to modify the inflammatory response.

#### 5.2.3 Mitigation of genotoxicity induced by different agents

Genotoxicity resulting from high exposure to genotoxic agents, of which the most important are mutagenic or carcinogenic chemicals and ionizing radiation, can promote DNA alterations and ultimately increase the risk of cancer development. Previous studies have demonstrated that in many cells (including rat liver and human lymphocytes), lycopene acts to prevent oxidative DNA damage [91,92]. For Srinivasan et al. [93], lycopene can be considered a natural protector against γ-radiation induced DNA damage, lipid peroxidation, and antioxidant status in primary culture of isolated rat hepatocytes. Moreover, Scolastici et al. [91] found that lycopene reduced the genotoxic effects of the agents when treated CHO cells with different genotoxic agents such as methylmethanesulphonate, H_2_O_2_, and 4-nitroquinoline-1-oxide.

In summary, lycopene shows great promise as a preventative agent in reducing the risk of prostate cancer. In addition to this aspect, the different protective properties of this natural pigment against genotoxicity and hepatotoxicity make it an excellent candidate for therapeutic applications that aim to protect liver cells but also to prevent specific harmful genetic alterations.

## 6 Effect of TP on physicochemical properties of food products

### 6.1 Effect of TP on physicochemical properties of bakery products

Recently, the trends in valorizing industrial by-products led to the formulation of new food products [94,95]. Using by-products from tomatoes in substituting or replacing raw materials enriches the newly formulated food products in bioingredients. The effects of the partial substitution of wheat flour with tomato seed powder or tomato pomace powder (TPP) on the physical and chemical characteristics of bakery products are shown in Table 5. The partial replacement of wheat flour with TPP in bakery products increased the chemical composition (moisture, crude protein, lipids, ash, and crude fiber) [16,[96], [97], [98], [99], [100]], mineral content (i.e., K, Ca, Mg, Na, Mn, Fe, and Zn), dietary fiber content, polyphenolic and tetraterpenoid compounds of these food products [16,99,100]. In contrast, the carbohydrate content decreased with increasing the level of substitution [97,99,100]. Cream crackers made from wheat flour and TPP showed the same tendencies with increasing carotenoids and tocols content [19]. Moreover, Mironeasa and colleagues [98] reported that in the composite flours made from wheat flour and tomato seed flour, the fat, protein, and ash increased as the level of tomato seed flour increased, whereas the moisture content decreased. Refined wheat flour partially substituted with 35 and 40 % TP in bread and muffins increased dietary fiber, vitamin C, antioxidant activity, and mineral content (Na, K, Mg, Ca, Fe) [37].

**Table 5:** Proximate, chemical and bioactive compositions of TP-formulated products.

Food products	Biscuits					Cake					Crackers					Bread		Muffin		Reference s
	Substitution level (%)					Substitution level (%)					Substitution level (%)					Substitution level (%)		Substitution level (%)		
	0	2.5	5	7.5	10	0	2.5	5	7.5	10	0	4	6	8	10	0	35	0	40	[ [16,19,37,99,100]]
**Proximate composition (g/100g)**																				
Moisture	3.69	4.51	5.30	6.14	6.97	21.75	22.57	23.35	24.21	25.04	n.d	n.d	n.d	n.d	n.d	33.2	34	32	35.9	
Crude protein	19.19	19.71	20.23	20.75	21.27	15.59	15.97	16.35	16.73	17.11	n.d	7.63	n.d	7.80	n.d	7.22	7.52	3.5	4.08	
Crude Fat	30.10	30.29	30.47	30.66	30.84	20.32	20.61	20.90	21.19	21.48	n.d	16.75	n.d	17.62	n.d	n.d	n.d	n.d	n.d	
Ash	0.89	0.96	1.02	1.09	1.15	1.57	1.63	1.68	1.74	1.80	n.d	1.52	n.d	1.55	n.d	n.d	n.d	n.d	n.d	
Crude fiber	3.60	3.80	4.00	4.19	4.39	4.49	4.69	4.87	5.08	5.25	n.d	n.d	n.d	n.d	n.d	2.00	4.05	2.99	3.52	
Carbohydrate	3.60	3.80	4.00	4.19	4.39	58.03	57.10	56.20	55.26	54.36	n.d	n.d	n.d	n.d	n.d	n.d	n.d	n.d	n.d	
**Mineral composition (mg/100g)**																				
Ca	24.44	24.49	24.53	24.64	24.87	40.07	40.12	40.17	40.22	40.29	n.d	36.62	n.d	49.39	n.d	22.39	27.45	11.30	14.19	
Zn	0.57	0.61	0.65	0.68	0.72	0.12	0.16	0.20	0.23	0.27	n.d	1.1	n.d	1.78	n.d	n.d	n.d	n.d		
Fe	1.43	1.49	1.54	1.60	1.65	0.95	1.00	1.06	1.12	1.17	n.d	1.26	n.d	1.58	n.d	2.50	2.90	1.49	3.48	
Mg	22.50	23.06	23.62	24.17	24.73	14.09	14.65	15.21	15.76	16.32	n.d	28.39	n.d	30.61	n.d	37.18	44.93	25.50	28.62	
K	16.36	17.09	17.82	18.55	19.28	22.61	23.34	24.07	24.80	25.53	n.d	210.8	n.d	273.8	n.d	133.74	197.61	71.53	100.39	
Na	38.87	39.96	41.05	42.14	43.23	68.53	69.62	70.71	71.80	72.89	n.d	n.d	n.d	n.d	n.d	647.94	615.84	381.37	461.40	
Mn	0.53	1.04	1.55	2.07	2.58	0.21	0.72	1.23	1.75	2.26	n.d	1.23	n.d	1.44	n.d	n.d	n.d	n.d	n.d	
**Dietary fiber (g/100g)**																				
Total dietary fiber	1.84	2.66	3.48	4.31	5.14	2.65	3.47	4.29	5.12	5.94	n.d	n.d	n.d	n.d	n.d	n.d	n.d	n.d	n.d	
Soluble dietary fiber	0.70	0.79	0.87	0.96	1.05	0.86	0.95	1.03	1.12	1.22	n.d	n.d	n.d	2.01	n.d	n.d	n.d	n.d	n.d	
Insoluble dietary fiber	1.14	1.87	2.61	3.35	4.09	1.79	2.52	3.26	4.00	4.72	n.d	3.48	n.d	4.67	n.d	n.d	n.d	n.d	n.d	
Total phenolic content	36.52	56.88	77.25	96.84	117.96	72.34	92.70	113.06	133.43	154.79	n.d	68.59	n.d	104.40	n.d	n.d	n.d	n.d	n.d	
Total flavonoid content	28.45	38.76	49.08	60.02	68.45	51.40	61.72	72.03	81.89	92.66	n.d	n.d	n.d	n.d	n.d	n.d	n.d	n.d	n.d	
**Fatty acids (%)**																				
Palmitic	n.d	n.d	n.d	n.d	n.d	n.d	n.d	n.d	n.d	n.d	20.28	20.54	20.58	20.88	21.35	n.d	n.d	n.d	n.d	
Stearic	n.d	n.d	n.d	n.d	n.d	n.d	n.d	n.d	n.d	n.d	4.16	4.18	4.54	4.77	4.92	n.d	n.d	n.d	n.d	
oleic	n.d	n.d	n.d	n.d	n.d	n.d	n.d	n.d	n.d	n.d	31.09	32.04	32.06	32.19	33.10	n.d	n.d	n.d	n.d	
Linoleic	n.d	n.d	n.d	n.d	n.d	n.d	n.d	n.d	n.d	n.d	38.60	39.24	39.31	40.66	41.74	n.d	n.d	n.d	n.d	
MUFA	n.d	n.d	n.d	n.d	n.d	n.d	n.d	n.d	n.d	n.d	33.52	33.99	34.28	33.92	33.75	n.d	n.d	n.d	n.d	
PUFA	n.d	n.d	n.d	n.d	n.d	n.d	n.d	n.d	n.d	n.d	40.64	40.41	40.21	40.78	40.94	n.d	n.d	n.d	n.d	
SFA	n.d	n.d	n.d	n.d	n.d	n.d	n.d	n.d	n.d	n.d	25.83	25.62	25.53	25.52	25.35	n.d	n.d	n.d	n.d	
**Carotenoid composition (mg/k)**																				
Lutein	n.d	n.d	n.d	n.d	n.d	n.d	n.d	n.d	n.d	n.d	0.53	0.64	0.59	0.57	0.59	n.d	n.d	n.d	n.d	
Zeaxanthin	n.d	n.d	n.d	n.d	n.d	n.d	n.d	n.d	n.d	n.d	0.02	0.04	0.04	0.04	0.05	n.d	n.d	n.d	n.d	
15-cis-neurosporene	n.d	n.d	n.d	n.d	n.d	n.d	n.d	n.d	n.d	n.d	nd	2.24	2.46	2.77	3.47	n.d	n.d	n.d	n.d	
δ-carotene-isomer	n.d	n.d	n.d	n.d	n.d	n.d	n.d	n.d	n.d	n.d	nd	1.56	1.61	1.63	1.69	n.d	n.d	n.d	n.d	
*trans*-lycopene	n.d	n.d	n.d	n.d	n.d	n.d	n.d	n.d	n.d	n.d	nd	2.35	2.49	2.53	2.76	n.d	n.d	n.d	n.d	
Tocols composition (mg/kg)																n.d	n.d	n.d	n.d	
α-tocopherol	n.d	n.d	n.d	n.d	n.d	n.d	n.d	n.d	n.d	n.d	18.52	21.40	23.65	24.10	25.46	n.d	n.d	n.d	n.d	
α-tocotrienol	n.d	n.d	n.d	n.d	n.d	n.d	n.d	n.d	n.d	n.d	7.76	9.10	8.83	8.64	8.81	n.d	n.d	n.d	n.d	
β-tocopherol	n.d	n.d	n.d	n.d	n.d	n.d	n.d	n.d	n.d	n.d	2.53	2.05	3.03	2.25	2.24	n.d	n.d	n.d	n.d	
β-tocotrienol	n.d	n.d	n.d	n.d	n.d	n.d	n.d	n.d	n.d	n.d	13.21	14.51	14.15	14.47	14.36	n.d	n.d	n.d	n.d	
γ-tocopherol	n.d	n.d	n.d	n.d	n.d	n.d	n.d	n.d	n.d	n.d	nd	2.17	2.80	3.94	4.55	n.d	n.d	n.d	n.d	
δ-tocopherol	n.d	n.d	n.d	n.d	n.d	n.d	n.d	n.d	n.d	n.d	15.57	16.09	15.43	15.20	15.02	n.d	n.d	n.d	n.d	
δ-tocotrienol	n.d	n.d	n.d	n.d	n.d	n.d	n.d	n.d	n.d	n.d	nd	0.90	1.55	1.60	1.19	n.d	n.d	n.d	n.d	
**Phenolic composition (mg/kg)**																				
4OH-benzoic acid der	n.d	n.d	n.d	n.d	n.d	n.d	n.d	n.d	n.d	n.d	87.60	94.78	90.92	91.06	92.21	n.d	n.d	n.d	n.d	
Coumaric acid	n.d	n.d	n.d	n.d	n.d	n.d	n.d	n.d	n.d	n.d	n.d	0.40	0.57	0.74	0.92	n.d	n.d	n.d	n.d	
Coumaric acid der	n.d	n.d	n.d	n.d	n.d	n.d	n.d	n.d	n.d	n.d	n.d	3.09	4.27	5.52	6.35	n.d	n.d	n.d	n.d	
Ferulic acid	n.d	n.d	n.d	n.d	n.d	n.d	n.d	n.d	n.d	n.d	2.55	4.30	4.28	4.44	5.13	n.d	n.d	n.d	n.d	
Gallic acid	n.d	n.d	n.d	n.d	n.d	n.d	n.d	n.d	n.d	n.d	9.93	70.99	124.81	160.65	173.12	n.d	n.d	n.d	n.d	
Naringenin	n.d	n.d	n.d	n.d	n.d	n.d	n.d	n.d	n.d	n.d	n.d	2.96	5.20	6.98	7.69	n.d	n.d	n.d	n.d	
Naringenin der	n.d	n.d	n.d	n.d	n.d	n.d	n.d	n.d	n.d	n.d	n.d	0.73	1.28	1.84	2.39	n.d	n.d	n.d	n.d	
Quercitin der	n.d	n.d	n.d	n.d	n.d	n.d	n.d	n.d	n.d	n.d	n.d	1.86	3.23	4.15	5.00	n.d	n.d	n.d	n.d	
Rutin	n.d	n.d	n.d	n.d	n.d	n.d	n.d	n.d	n.d	n.d	n.d	9.81	17.26	23.76	30.20	n.d	n.d	n.d	n.d	

The values are given on the dry weight basis, n.d = not determined.

Padalino and colleagues [101] investigated the impact of incorporating different particle sizes of tomato peels on durum wheat whole-meal spaghetti. The results showed that fine particles of tomato peels help to enhance the spaghetti quality. While peel particle size did not affect protein and available carbohydrate content, the fine particles induced significant increments of tetraterpenoids in formulated samples. The dietary fiber content of samples supplemented with fine particles is less than that of samples supplemented with coarse. Also, the results of *in vitro* starch digestibility suggested that particle size influenced the starch digestibility, which decreases in spaghetti enriched with coarse particle size.

### 6.2 Effect of TP on dough rheological behavior, physicochemical characteristics of composite dough of bakery products

Incorporating tomato seed flour (TSF) in dough formulations influenced dough rheological properties (Table 6). Indeed, the dough extensibility, index of swelling, and baking strength decrease when the TSF level increases [97]. Likewise, replacing wheat flour with tomato seeds flour by up to 10 % increased the bread samples' loaf volume, porosity, and elasticity [97]. Otherwise, wheat flour supplemented at 6 and 10 % levels (w/w flour basis) increased bread crumb elasticity and reduced specific volume and bread crumb porosity [102]. TP added increased the crumb firmness, resulting in a lower hardness value in tomato-based bread and muffins. Furthermore, incorporating TSF in the dough also increased the chewiness, cohesiveness, and springiness [37]. Mironeasa and colleagues [98] concluded that the partial replacement of refined wheat flour by TSF strengthens the dough system of bakery products.

**Table 6:** Dough rheological behavior, physicochemical characterization of composite dough or products obtained with wheat and tomato seed flours or TP powder.

Characteristics	Bread					Muffin		Cracker					Sources
	Substitution level (%)			Substitution level (%)		Substitution level (%)		Substitution level (%)					
	0	6	10	0	35	0	40	0	4	6	8	10	[ [19,37] [102]]
Specific volume (cm^3^/g)	5.18	4.84	4.81										
Bread crumb porosity (%)	85.28	84.24	84.15										
Bread crumb Elasticity (%)	89.23	92.18	90.77										
Hardness (N)				3.68	0.011	70.82	47.14						
Adhesiveness (Ns)				0.23	0.27	0.40	0.59						
Springiness				0.87	0.99	0.98	1.62						
Cohesiveness				0.72	0.77	0.71	0.99						
Gumminess				1.17	1.34	50.79	46.88						
Chewiness				4.58	5.32	50.03	96.49						
Peak viscosity (BU)								895.0	898.5	946.0	971.0	1024.0	
Breakdown								297.0	308.0	345.0	390.0	422.5	
Final viscosity (BU)								1042.5	1047.5	1058.0	1068.0	1147.0	
Pasting temperature (°C)								60.7	61.3	61.3	61.5	61.8	

BU: Brabender Units.

### 6.3 Effect of TP on meat by-products

Recently, meat by-products have been formulated by replacing the fat content with different levels of TP (Table 7). For instance, tomato peels were incorporated in beef burgers and sausages up to 15 % [103], resulting in decreased fat levels, and contrast, protein, ash, and fiber levels increased in the end products. Consequently, the energy value that beef burgers and sausages should provide decreased significantly [103]. This result shows that TP is a suitable coproduct for formulating low-calorie foods. In the same study, it was figured out that the introduction of tomato peels in meat products showed a positive effect on the cooking yield, reducing the loss during the cooking decreased with increasing the level of tomato peels. The decrease in the cooking loss may be due to the high fiber content of tomato peels, which bound more water [103]. This result agrees with that reported by Namir and colleagues, who found that beef burgers formulated by partially replacing the fat level with tomato fiber pectin (TFP) showed a decrease in cooking loss and a reduction in the diameter of burgers [104]. In addition, Yadav and colleagues showed that adding tomato peel to chicken sausages decreased moisture content and increased ash and crude fiber contents [105]. Moreover, the emulsion stability and cooking yield were significantly higher in the sausages containing 6 and 9 % tomato peels [105].

**Table 7:** Effect of tomato peels as a fat replacer on the chemical composition of burger and sausage.

Products	Beef burger				Beef sausage				Chicken sausages				Sources
Component (%) on a dry weight basis	Substitution level (%)				Substitution level (%)				Substitution level (%)				
	0	5	10	15	0	5	10	15	0	3	6	9	[103] [105]
Moisture	0.00	0.00	0.00	0.00	0.00	0.00	0.00	0.00	68.71	67.66	66.78	64.40	
Crude protein	41.50	41.94	42.50	43.13	42.25	42.48	42.96	43.16	173	169	167	166	
Ether extract/fat	32.25	31.51	30.69	30.12	34.98	32.44	31.86	30.23	89	85	82	82	
Ash	4.05	4.20	4.53	4.76	3.39	3.58	3.88	4.07	2.2	2.4	2.5	2.6	
Crude fiber	3.00	4.15	4.89	5.79	2.00	4.25	6.50	8.72	0.2	1.1	1.9	2.7	
Total carbohydrates	22.20	22.35	22.28	21.96	19.38	21.50	21.30	22.54					
Available carbohydrates	19.20	18.20	17.39	16.17	17.38	17.25	14.80	13.82					

### 6.4 Effect of TP on snacks' nutritional quality

Snacks supplemented with antioxidant and dietary fiber sources, such as TP powder or other fruit pomace powder, have been formulated to improve their nutritional and functional properties. Generally, the partial replacement of wheat flour with fruit pomace powder increases snacks' physicochemical, nutritious, and functional characteristics. For example, adding tomato seed meal to tarhana increased its protein, oil, insoluble dietary fiber, total dietary fiber, ash, mineral, and total phenolic contents and antioxidant activity [16]. In addition, the incorporation of tomato seed increased tarhana's lysine, phenylalanine, threonine, serine, alanine, glycine, histidine, aspartic acid, arginine, and tyrosine contents [16]. Similarly, El-Araby reported that the snacks' fortification with apple pomace powder and TPP increased its contents in protein, ash, fiber, calcium, potassium, and phenolic compounds [106].

## 7 Sensory liking of TP-based food products

Due to its natural pigment (carotenoid and polyphenolic) contents, color is one of the primary sensory effects induced by TP, especially tomato peels, on food products containing them. Generally, the TP addition of the food product results in a decrease in lightness (L* color value), while the redness (a* color value) and yellowness (b* color value) significantly increased [20,37,107]. A study showed that ice creams containing 2 % and 3 % carotenoids extracted from tomato peels had the best sensory characteristics in terms of color, aroma, body, and texture, melting and mixing compared to ice creams enriched with 1 %, 4 %, and 5 % of the extract. Furthermore, the enrichment of the sausage by the TP leads to an improvement in the color attractiveness of the final products [108]. These authors say TP positively affects consumer liking, including acceptability and preference. Indeed, substituting 6 % dry TP in refined wheat flour resulted in bread with good sensory properties. The overall acceptability was also good with this percentage, but when the substitution of dry TP was increased to 10 %, the bread obtained had a fair acceptability [102]. However, a recent investigation reported that extruded snacks containing 10 % TP performed the best scores in terms of physical and sensory characteristics [20]. This shows that the level of TP fortification and acceptance of the final product varies depending on the food matrix.

Moreover, a study by Concha-Meyer and colleagues (2019) assessed consumers' sensory appreciation and purchase intention for bread containing different portions (0, 5, and 10 %) of TPP. According to the panelists, the sample containing 5 % TP had a mild and pleasant aftertaste compared to the sample containing 10 % TP. The former had similar sensory characteristics to the control. The weak score obtained by the latter had been reported by Isik & Tokpaya [16], who used 12 % of TP in the cracker. This bitter aftertaste would be due to furostanol saponin and probably the higher phenolic compound content in the products containing more TP powder. According to these authors, consumer interest in health and sensory liking significantly affects the product's purchase intent. In addition, the relatively low cost of the TP product is a comparative advantage for functional food consumption. Moreover, several researchers [16,20,102] reported that incorporating up to 10 % of TP in baked goods and related products did not jeopardize its sensory acceptability.

## 8 Shelf life extension induced by TP

Extending the shelf life of food products is one of the main advantages of using TP in food applications. Adding TP (TP, TP constituent, or extract from both) at various doses in diverse food matrices has shown natural preservative effects on the final product by prolonging its shelf life (Table 8). For instance, the study of Mehta and colleagues (2018) showed that the partial substitution of refined wheat with 35 % and 40 % of TP in muffins and bread had extended their shelf life, regardless of the storage temperature. Compared to the control, the shelf life was lengthened from 24 to 72 h, depending on the treatment. In addition, replacing TP below 5 % in Barbari bread leads to delaying staling during the storage period (24–96 h) at 25 °C [109]. The bioactive compounds of TP, especially lycopene, β-carotene, polyphenols, and tetraterpenoids, while protecting against oxidative degradation, exert inhibition effects against spoilage microbes. As a result, the shelf life of final products containing TP increases significantly. These protective effects allowed the TP extract used at 400 mg/kg to extend the shelf life of traditional Tunisian butter to 60 days when stored at 4 °C without compromising the aroma development [17]. Similarly, the study by Andres and colleagues (2016) revealed that applying organic TP extracts on the surface of lamb meat extended its shelf life by 7 days [110]. Moreover, TP has shown a significant thixotropic effect on ketchup and has demonstrated its ability to improve the thermal stability of food products during heat processing [111].

**Table 8:** Effect of TP on shelf life extension of food products.

Food products	TP constituent	Incorporated Dose/percentage	Main outcome related to shelf life extent	References
Traditional Tunisian butter (TTB)	TP extract	400 mg of extract/kg	Protective effect against lipid peroxidation, prolongation of shelf-life up to 60 days	[17]
Bread and muffin	TP	35–40 % in bread and muffin, respectively.	Increase the dietary fiber, vitamin C, antioxidant activity and minerals, prolongation of shelf-life up to 8 days and 12 days for bread and muffin, respectively	[37]
Meat longissimus thoracis	TP extract	3 g of TP extract/36.13 ± 2.85 kg	Increase of potential antioxidant agents like lycopene, phenol and β-carotene contents, Prolongation of shelf-life up to 7 days	[110]
Bread	TP	10 % tomato seed/100 g	Color intensity, expressed as the L*, a*, b* values of breads	[121]
Barbari bread	TP	≤5 %	Delays staling during storage at 25 °C for 1–4 days	[109]
Bread	TP	10 % dry tomato/100 g	Increase in moisture content, titratable acidity, and breadcrumb elasticity, reduction in specific volume and bread crumb porosity	[102]
Raw Fermented Sausages	freeze-dried TP	1.5 % freeze-dried TP/100 g	Increase in antioxidant activity, reduced levels of nitrites, improved sausage shelf-life stability	[115]
Snack	TP	10 % tomato pomace/100 g	Increase antioxidant activity, high storage stability	[122]
Bread	TP	7 % tomato pomace power/100 g	Increase dough water absorption, reduction in dough arrival, development, and stability times, and increase dough softening after 5 and 12 min.	[109]

## 9 Challenges and future trends

Research endeavors focused on utilizing agro-industrial by-products, such as fruit and vegetable pomace, as valuable constituents in food production hold substantial promise. This area of inquiry stands poised to make noteworthy contributions to enhancing food quality and the security of nutritional resources. TP is a rich source of minerals (K, Mg, Ca, P, Na, and Fe) and various vitamins, including C, E, and carotenoids, as the primary source of provitamin A. With this micronutrient and phytochemical contents, TP can be used as a cheap and valuable functional ingredient in addressing the most common micronutrient deficiencies in children under five, including iron, zinc, calcium, phosphorus, and vitamin A, in developing countries. However, its possible use as a fortifier in infant flour formulation must consider its high dietary fiber content since, according to Belović et al. [111], it can contain up to 60 % (on a dry matter basis). Lu and colleagues have recently demonstrated that yeast fermentation improves the bioaccessibility of nutrients and bioactive compounds in tomato juice [112]. This low-cost bioprocess can be used in increasing TP nutrient bioaccessibility, mainly when it is used in infant food formulation. Another essential food that TP could enrich is Gari. It is one of the most popular staple foods in Sub-Saharan Africa, made from cassava. It is a traditional food high in carbohydrates and deficient in micronutrients and other macronutrients [113]. Gari is consumed by both youth and adults. Therefore, fortification of gari with TP would be an effective way to combat nutritional insecurity in this region. Developing specific low-calorie food products could be another form of food valorization of TP.

Lipid oxidation and microbial degradation are the two significant biological phenomena threatening the stability of food products during storage. TP contains various phytochemical compounds that exhibit considerable antioxidant activities. In addition, research has shown that TP has antimicrobial properties that reduce many spoilage microorganisms [14,15,114,115]. Consequently, TP is also an essential ingredient for preserving certain food products. Thus, TP could be an excellent functional ingredient for improving foods' nutritional and sensory quality and their natural preservation during storage. Traditional fermented condiments from Africa, such as African locust bean-based condiments (*Afitin, iru, soumbala, nététu*) and African fermented fish (*Lanhouin, momone, Guedj Jalan Mindeshi Dagaa Adjonfa*) have a short shelf life due to lipid oxidation and lack of controlling microbial spoilage [113,116,117]. Therefore, preservation trials of these traditional fermented condiments with TP could be envisaged. Moreover, the study on the bioactive compounds of TP, in particular dietary fiber, is quite limited. Considering the mass importance of this constituent (60 %), its technological interest and its impact on the health of consumers must be dug into more deeply. Several studies have pointed out valorization of food wastes and by-products as a solution to improve the economic and environmental sustainability of the food production. Numerous valorization schemes have been proposed to explore food wastes and by-products as biomass suppliers to obtain different bio-based products. However, indication of the main challenges to guarantee the employment of TP in the food industry, should point out.

## 10 Conclusion

Thisreview updated and summarized the nutritional and bioactive properties and different food applications of TP. TP is a by-product of industrial tomato processing with nutritious and bioactive content. Its use as a food ingredient in relevant doses in various food products has enhanced its nutritional quality and sensory and functional properties and extended its shelf life. However, further work should address the effect of dietary fiber content in TP on the bioaccessibility of other bioactive compounds. In addition, the extraction and optimization of lycopene from TP peels using natural deep eutectic solvents and its use as a food colorant in replacement synthetic dyes should be explored.

## Data availability statement

Data will be made available on request.

## CRediT authorship contribution statement

**Ifagbémi Bienvenue Chabi:** Writing – original draft, Visualization, Software, Methodology, Investigation, Data curation, Conceptualization. **Oscar Zannou:** Writing – original draft, Visualization, Software, Methodology, Investigation, Data curation. **Emmanuelle S.C.A. Dedehou:** Writing – original draft, Visualization, Investigation, Data curation. **Bernolde Paul Ayegnon:** Writing – original draft, Visualization, Investigation, Data curation. **Oloudé B. Oscar Odouaro:** Writing – original draft, Visualization, Investigation, Data curation. **Sajid Maqsood:** Writing – review & editing, Validation, Resources, Methodology, Investigation. **Charis M. Galanakis:** Writing – review & editing, Validation, Resources, Methodology, Investigation. **Adéchola Pierre Polycarpe Kayodé:** Writing – review & editing, Validation, Resources, Methodology, Investigation.

## Declaration of competing interest

The authors declare that they have no known competing financial interests or personal relationships that could have appeared to influence the work reported in this paper.
